# Concurrent Preimplantation Genetic Testing and Competence Assessment of Human Embryos by Transcriptome Sequencing

**DOI:** 10.1002/advs.202309817

**Published:** 2024-06-20

**Authors:** Yuqian Wang, Ye Li, Xiaohui Zhu, Ming Yang, Yujun Liu, Nan Wang, Chuan Long, Ying Kuo, Ying Lian, Jin Huang, Jialin Jia, Catherine C. L. Wong, Zhiqiang Yan, Liying Yan, Jie Qiao

**Affiliations:** ^1^ State Key Laboratory of Female Fertility Promotion Center for Reproductive Medicine Department of Obstetrics and Gynecology Peking University Third Hospital Beijing 100191 China; ^2^ National Clinical Research Center for Obstetrics and Gynecology (Peking University Third Hospital) Beijing 100191 China; ^3^ Key Laboratory of Assisted Reproduction (Peking University) Ministry of Education Beijing 100191 China; ^4^ Beijing Key Laboratory of Reproductive Endocrinology and Assisted Reproductive Technology Beijing 100191 China; ^5^ National Clinical Key Specialty Construction Program Beijing 100191 China; ^6^ Peking‐Tsinghua Center for Life Sciences Peking University Beijing 100871 China; ^7^ Academy for Advanced Interdisciplinary Studies Peking University Beijing 100871 China; ^8^ Peking‐Tsinghua Center for Life Sciences Tsinghua University Beijing 100084 China

**Keywords:** implantation, monogenic defects, preimplantation genetic testing, transcriptome

## Abstract

Preimplantation genetic testing (PGT) can minimize the risk of birth defects. However, the accuracy and applicability of routine PGT is confounded by uneven genome coverage and high allele drop‐out rate from existing single‐cell whole genome amplification methods. Here, a method to diagnose genetic mutations and concurrently evaluate embryo competence by leveraging the abundant mRNA transcript copies present in trophectoderm cells is developed. The feasibility of the method is confirmed with 19 donated blastocysts. Next, the method is applied to 82 embryos from 26 families with monogenic defects for simultaneous mutation detection and competence assessment. The accuracy rate of direct mutation detection is up to 95%, which is significantly higher than DNA‐based method. Meanwhile, this approach correctly predicted seven out of eight (87.5%) embryos that failed to implant. Of six embryos that are predicted to implant successfully, four met such expectations (66.7%). Notably, this method is superior at conditions for mutation detection that are challenging when using DNA‐based PGT, such as when detecting pathogenic genes with a high de novo rate, multiple pseudogenes, or an abnormal expansion of CAG trinucleotide repeats. Taken together, this study establishes the feasibility of an RNA‐based PGT that is also informative for assessing implantation competence.

## Graphical abstract

The accuracy and applicability of routine preimplantation genetic testing (PGT) is confounded by uneven genome coverage and a high allele drop‐out rate from single‐cell whole genome amplification. This study proposes an alternative method to PGT by leveraging the abundant mRNA transcript copies present in trophectoderm cells to accurately diagnose genetic mutations and concurrently evaluate embryo competence.

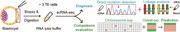

## 1 Introduction

Over 8000 monogenic disorders have been documented in the Online Mendelian Inheritance in Man (OMIM, http://omim.org) database. Although monogenic disorders are considered rare, collectively, their prevalence is up to 1% of the population.^[^ ^1^ ^]^ Moreover, the vast majority of monogenic disorders manifest in infants and children, are often associated with lifelong disability and mortality, and are currently without effective treatments.^[^ ^2^ ^]^ Nevertheless, with the rapid developments in the fields of clinical and molecular genetics over the last decades of research, the biological bases for as many as 6500 monogenic disorders have now been defined.^[^ ^3^ ^]^ For families with identified pathogenic variants or a history of disorders, or both, preimplantation genetic testing (PGT) should provide the ability to block the transmission of disease to the next generation.^[^ ^4^ ^]^

PGT has been widely utilized in the clinic for the detection of monogenic/single‐gene defects (PGT‐M) since it was first introduced in the 1990s, using polymerase chain reaction (PCR).^[^ ^5^, ^6^, ^7^, ^8^ ^]^ Multiplex PCR and fluorescence in situ hybridization (FISH) were subsequently introduced as a high‐throughput screening approach for numerical and structural chromosomal abnormalities.^[^ ^9^, ^10^ ^]^ Over the last decade, genome‐wide technologies and strategies, including next‐generation sequencing (NGS) and single nucleotide polymorphism (SNP) array based on single‐cell whole genome amplification (WGA), have been used to perform aneuploidy analysis and mutation diagnosis in PGT‐M.^[^ ^11^ ^]^

For embryonic mutation diagnosis by PGT‐M, whole genome analysis is followed by PCR‐based direct mutation detection and short tandem repeats (STR)/SNP‐based linkage analysis.^[^ ^10^, ^11^ ^]^ WGA methods such as multiple displacement amplification (MDA) and multiple annealing and looping‐based amplification cycles (MALBAC) are utilized because these lead to the preparation of an adequate amount of DNA for subsequent genetic testing. However, DNA amplification failure and DNA contamination typically occur in ≈10% of samples. Moreover, uneven genome coverage and high allele drop‐out (ADO) rate (≈20%) are factors that affect the accuracy and applicability of PGT‐M.^[^ ^12^ ^]^ Although linkage analysis can improve diagnostic reliability and accuracy,^[^ ^11^, ^13^ ^]^ relevant family members with clinically relevant genetic variants are required to construct mutation‐linked haplotypes. While, linkage analysis is inapplicable for de novo mutations or in subjects that lack a positive family history.^[^ ^10^, ^11^ ^]^ For instance, Neurofibromatosis type 1 (NF1, prevalence 1:2500‐1:3000) is caused by mutations in the *NF1* gene and is a common neurocutaneous disorder with a de novo mutation rate of up to 50%.^[^ ^14^ ^]^ This high de novo mutation rate and the existence of multiple pseudogenes altogether presents a challenge for both linkage analysis and direct mutation detection in embryos.^[^ ^15^ ^]^

Previous studies have suggested that aneuploidy screening could increase implantation success rates, however DNA‐based PGT does not significantly raise the implant success and cumulative live birth rates of tested embryos.^[^ ^8^, ^16^, ^17^ ^]^ Currently, it is recognized that DNA‐based PGT does not predict the potential of embryos for implantation and developmental competence.^[^ ^18^, ^19^, ^20^ ^]^ However, extraembryonic tissues could also be significant for testing preimplantation embryo competence, as follows. The process of implantation involves the interaction between the outer cell layer of the blastocyst formed by trophectoderm (TE) and uterus endometrium that the embryo attaches to the endometrial epithelium and then invades the endometrial stroma.^[^ ^21^ ^]^ Trophoblast cells derived from TE have an important contribution to placenta and must maintain multipotency during implantation.^[^ ^22^, ^23^ ^]^ Our recent omics analysis revealed that the precise regulation of gene expression in the TE plays a key role in embryo implantation and subsequent development.^[^ ^24^ ^]^ Another omics study also proposed the possibility of assessing embryo competence by analysis of the TE transcriptome.^[^ ^25^ ^]^

Considering the limitations of DNA‐based PGT‐M, we posited that RNA‐based diagnosis may be a feasible alternative that could have comparable or even superior adaptability and accuracy of embryonic genetic testing. Moreover, the TE transcriptome could provide multi‐dimensional information on gene expression and molecular homeostasis of embryos, which altogether may offer additional insight when selecting viable embryos. In this study, we developed a transcriptome‐based approach to PGT‐M and tested its applicability with embryos from 26 families with monogenic disorders. Our study shows that TE transcriptome analysis is informative for mutation detection, linkage analysis and competence evaluation of preimplantation embryos. We also performed differential expression analysis of embryos from successful and failed implantations to identify a gene set that is highly correlated with implantation and development potential. Overall, our study suggests that an RNA‐based PGT‐M that analyses the TE transcriptome could be viable as a clinical diagnostic for detecting genetic mutations and evaluating embryo competence, especially for conditions presenting with high levels of candidate gene expression, low parental expression bias, problematic WGA‐coverage, or any combination of these factors that pose a challenge to DNA‐based PGT‐M.

## 2 Results

### 2.1 Feasibility Assessment of RNA‐based PGT

**Figure 1:**
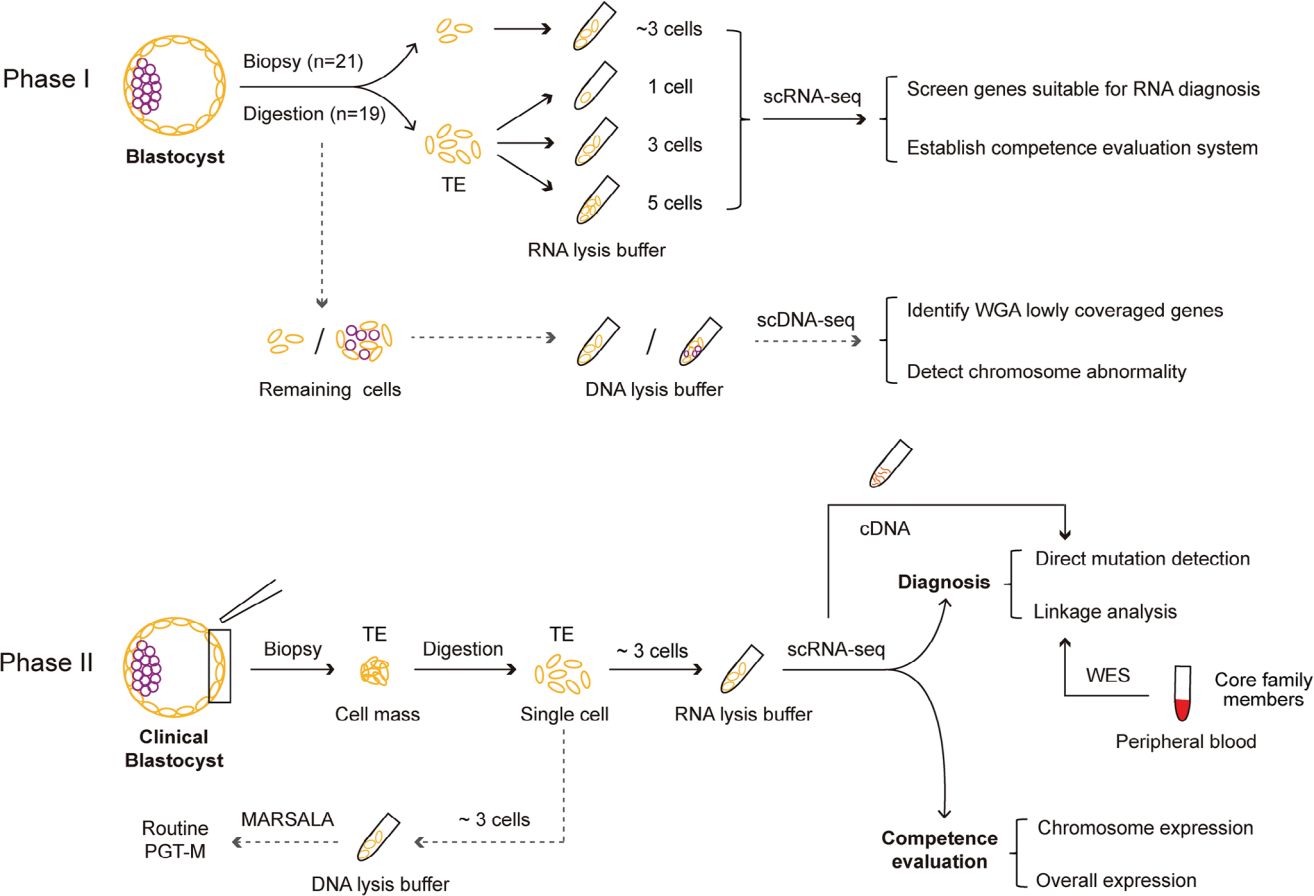
Schematic illustration of this study. (*Top*) Phase I, feasibility assessment of RNA‐based PGT. Nineteen donated blastocysts were digested, and the TE cells were divided into three groups containing 1, 3, and 5 cells, which were then subjected to RNA‐seq. Twenty‐one blastocysts were biopsied and ≈3 TE cells were collected for subsequent RNA‐seq. In parallel, DNA‐seq experiments were also performed for all blastocysts. The solid lines show the experiment and analysis pipelines based on RNA‐seq data. The dotted lines show the pipeline of DNA‐seq used for the comparison. (*Bottom*) Phase II, the application strategy for RNA‐based PGT in the clinic. The process comprises three parts: direct mutation detection, linkage analysis, and competence evaluation. The solid lines represent the RNA‐based PGT pipeline, and the dotted lines represent the routine PGT analysis used for diagnostic comparison.

To assess whether the TE transcriptome can be used for PGT, 19 donated whole blastocysts and 21 biopsied materials from 21 blastocysts were obtained from 20 couples. Nineteen donated blastocysts were digested, and the TE cells were divided into three groups containing 1, 3, and 5 cells, which were then subjected to RNA‐seq (**Figure** **1**; Table S1, Supporting Information). The remaining cells of digested blastocysts were collected for DNA‐seq as a comparison. Similarly, for biopsied TE material of the 21 blastocysts, ≈3 TE cells were selected for subsequent RNA‐seq, and the remaining TE cells were collected for DNA‐seq, which clinical outcomes were followed and summarized so as to establish an embryo competence evaluation system (Figure 1; Table S1, Supporting Information). A detailed description of the samples is provided in Table S1 (Supporting Information).

**Figure 2:**
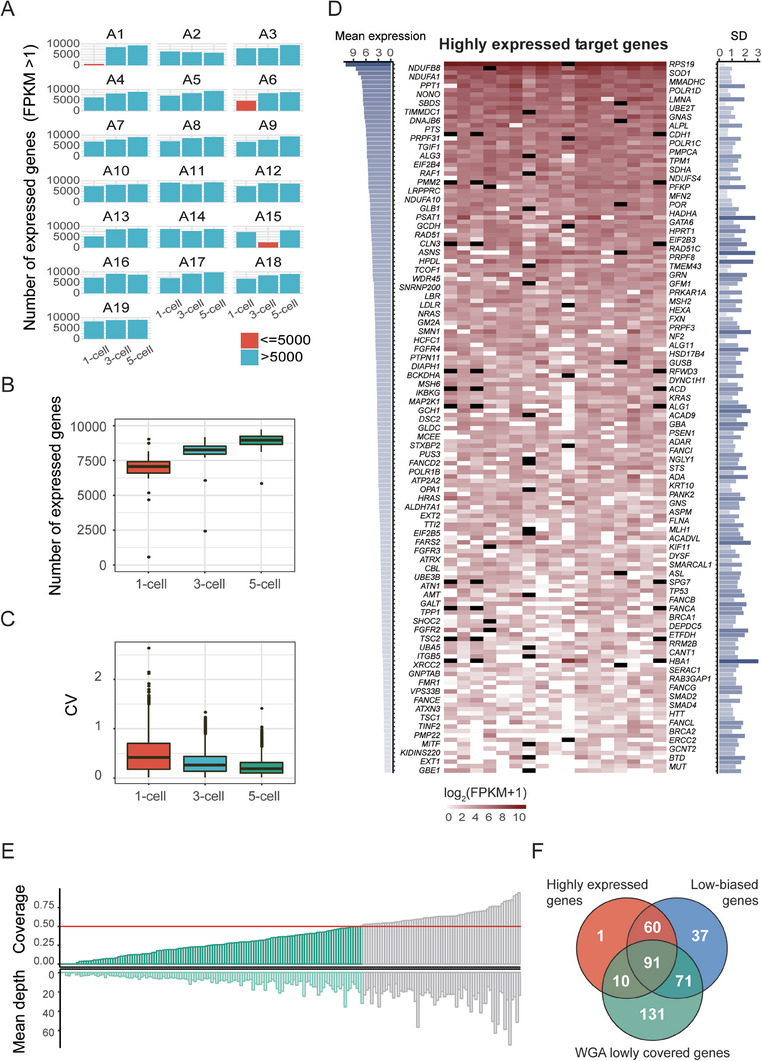
Experimental conditions for evaluating the feasibility of the RNA‐based PGT approach. A) The number of expressed genes in the TE samples that were collected from donated blastocysts. In each of the 19 donated blastocysts (A1‐A19), three sets of samples (containing 1 cell, 3 cells and 5 cells) were evaluated. The majority expressed over 5000 genes (FPKM > 1). B) Boxplot of the expressed gene number from 1‐cell, 3‐cell and 5‐cell groups. All three groups of samples expressed comparable numbers of genes. C) Boxplot showing the gene coefficients of variation from the 1‐cell, 3‐cell and 5‐cell groups. D) Overview of highly expressed genes (average log_2_ (FPKM+1) > 1.5) in TE cells. The average gene expression is shown, with darker colors representing higher expression levels (left). Gene expression pattern (shown as log_2_ (FPKM+1)) for 1‐cell samples, with gene names labeled on both sides along the y‐axis (middle). SD of each gene, with darker labels indicative of larger SD values (right). Black represents genes on aneuploid chromosomes, which are excluded in the expression statistics. E) Barplots showing the coverage and depth of disease‐causing genes in WGA (MALBAC) data. Genes with coverage lower than 0.5 (red line) were considered to be WGA low‐coverage genes. F) Venn diagram featuring highly expressed genes (shown in D), low‐biased genes (shown in Table S3, Supporting Information) and WGA low‐coverage genes (shown in E). Highly expressed genes with low parental expression bias and genome coverage are considered more suitable for RNA‐based PGT.

In total, 57 TE samples from 19 donated blastocysts (Table S1, Supporting Information) were initially processed to evaluate the feasibility of RNA‐based genetic testing. The number and stability of expressed genes could reflect the quality of TE cells and the clinical analysis repeatability. The overwhelming majority (54/57, 94.7%) of the TE samples expressed over 5000 genes, so we took this as the quality control (**Figure** **2**A,B). Gene expression levels were relatively stable among TE cells regardless of cell numbers sampled (Figure 2C). These properties are ideal for the accessibility and reproducibility of the transcriptome‐based method when applied to PGT.

Among 4868 Mendelian genetic disorders related genes that are documented in OMIM, 2814 genes were expressed in TE cells (Table S2, Supporting Information). According to the data from the European Society of Human Reproduction and Embryology (ESHRE) PGD Consortium,^[^ ^26^ ^]^ as well as data from our center, there are 527 PGT‐M target genes (Table S3, Supporting Information). Whether the TE transcriptome can be used to diagnose mutations in those target genes depends on both the expression level and expression variation of those genes. Genes with FPKM>1 have been shown to exhibit confident expression.^[^ ^27^ ^]^ Theoretically, variants in genes with FPKM>1 can be detected using single cell RNA‐seq or PCR. In this study, to obtain more accurate results, genes with log_2_(FPKM+1)>1.5 were considered as candidates for RNA‐based variant detection. Among these target genes, we found that 162 of them are relatively highly expressed in single TE cells (Table S4, Supporting Information). Besides, these genes can be detected in the vast majority of TE sample (Figure 2D), which suggests that RNA‐based PGT‐M is likely to be feasible to detect these genes for causative variants. The gene sets suitable for RNA‐based PGT‐M are similar regardless of the initial TE cell number of a sample (Figure S1A, Supporting Information). In addition to expression levels, parental expression bias is another important factor that influences the precision of PGT as a diagnostic tool. The parental expression bias was assessed through the SNP heterozygosity and 259 genes display parental expression (Table S4, Supporting Information). Moreover, we analyzed the coverage and depth of all target genes using data from single cell DNA‐seq (Figure 2E). Notably, genes with low genome coverage and depth were more prone to amplification failure and ADO, resulting in misdiagnosis of mutation detection. Through the above analysis, we identified 91 highly and stably expressed genes with low‐bias, and with low coverage and depth from WGA (Figure 2F; Table S4, Supporting Information). These results are consistent with the notion that TE transcriptome analysis is suitable for PGT and that the 91 screened genes are ideal candidates for RNA‐based PGT‐M analysis.

### 2.2 RNA‐based Clinical Genetic Testing Strategy

Our preliminary results established the feasibility of using the TE transcriptome to detect variants of target genes. Next, we recruited 26 families with 18 monogenic disorders mapping to 22 pathogenic genes (clinical information is shown in Table S5, Supporting Information). In total, 82 blastocysts from 26 IVF cycles were obtained and biopsied. After digestion of the biopsied TE mass, ≈3 cells were collected for RNA‐based genetic testing, while ≈3 other cells were subjected to routine DNA‐based PGT for comparison (Figure 1). The RNA reverse transcription products were used as templates for direct mutation detection. Among the families with whole exome sequencing (WES) data from core members, linkage analysis was also performed to confirm the mutant alleles. Furthermore, the competence evaluation system was established and utilized for implantation prediction (Figure 1).

### 2.3 Direct Mutation Detection Based on RNA Analysis

**Figure 3:**
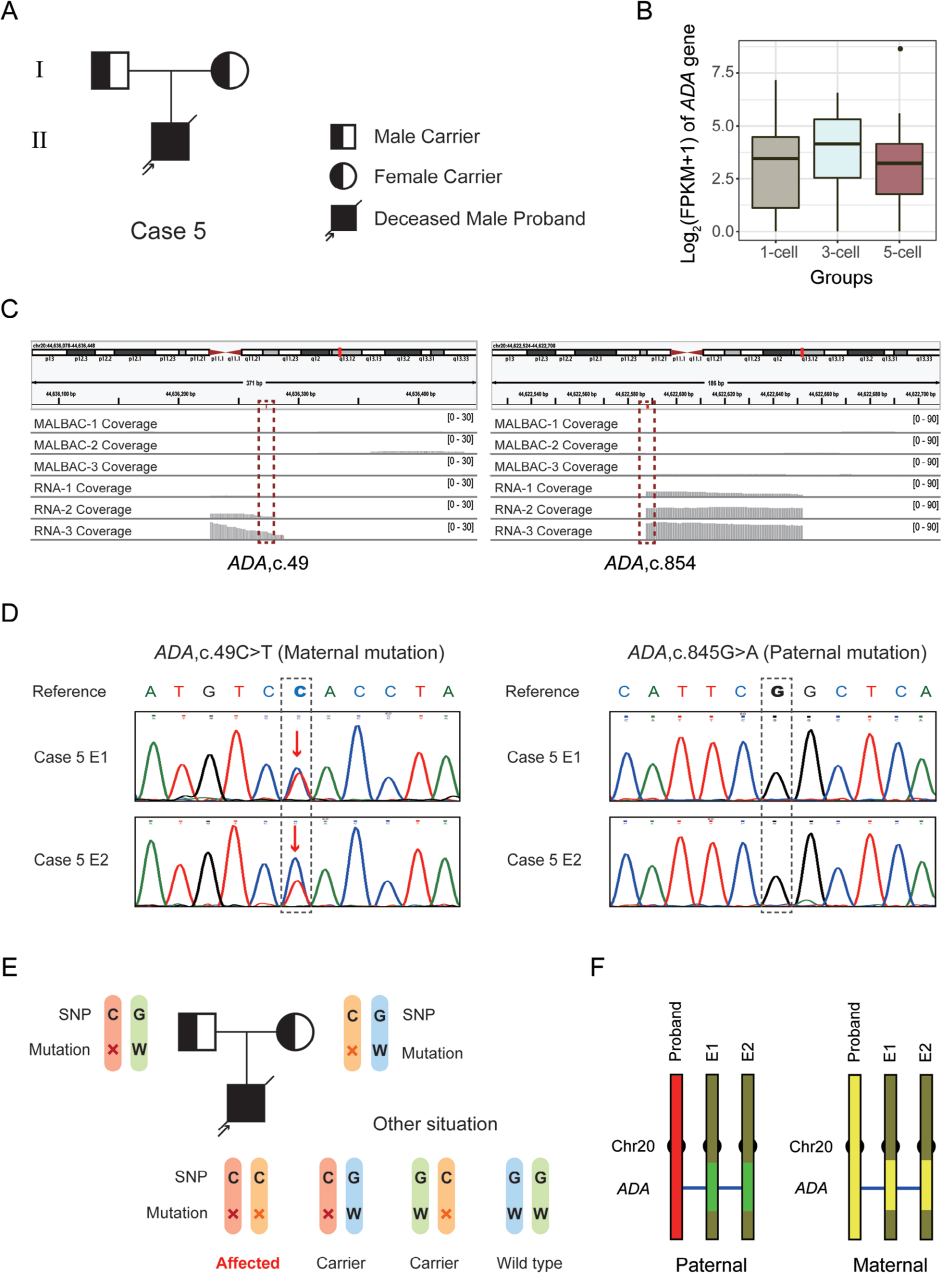
RNA‐based mutation diagnosis for case 5 with autosomal recessive SCID. A) Pedigrees of the SCID family. The filled symbol represents the affected patient, and the half‐filled symbol represents the carrier of this disease. Circle and square indicate female and male, respectively. The arrow indicates the affected proband. Diagonal line represents a deceased individual. B) *ADA* gene expression levels were assessed in three TE groups. The abscissa indicates the 1‐cell, 3‐cell and 5‐cell groups and the ordinate represents the gene expression level united by log_2_ (FPKM+1). C) IGV plot shows the coverage of *ADA* in DNA and RNA sequencing data from TE cells. The mutation loci are indicated by red dotted boxes. D) The direct mutation detection results of 2 embryos in case 5 determined by Sanger sequencing following PCR based amplification of RNA. Dotted boxes show the mutation loci, and the red arrows indicate the mutations. E) SNP analysis schematic of this autosomal recessive case. The symbol “×” indicates the mutation and the letter “W” represents the wild‐type. The SNP base “C” is linked with the mutation allele and “G” is linked with the wild‐type allele. Embryos carrying C/C inherited parental mutations while those carrying G/G inherited parental wild‐type alleles. Embryos carrying C/G inherited only paternal or maternal mutations, which are also called carriers. F) The SNP analysis results of 2 embryos using WES and transcriptome data. SNP markers within 10 Mb upstream/downstream around the mutations were analyzed and illustrated.

Direct mutation detection was performed for all 82 blastocysts on the basis of PCR using RNA reverse transcription products. Three cases containing 2 autosomal recessive families and 1 autosomal dominant family were taken as examples to illustrate the approach. In case 5, the proband was diagnosed as severe combined immunodeficiency disease (SCID). Genetic diagnosis of the proband showed compound heterozygous mutations c.49C>T (maternal) and c.845G>A (paternal) at the Adenosine Aminohydrolase (*ADA*) gene (**Figure** **3**A; Table S5, Supporting Information). *ADA* is highly and stably expressed (mean expression level, log_2_(FPKM+1) = 3.26) in TE cells (Figure 3B), whereas it shows no genomic coverage (coverage = 0) in WGA data (Figure 3C). We detected the mutations in the RNA reverse transcribed (cDNA) product in 2 embryos for this case. The two embryos both inherited the maternal mutation, exhibiting a bimodal C/T at c.49 site, and were free of the paternal mutation as determined by the presence of a wild‐type G/G at the c.845 site (Figure 3D and **Table** **1**).

**Table 1:** RNA‐based PGT results and clinical outcomes of 82 embryos from 26 families.

Case	Gene	Heredity model	Embryo ID	Cell number/ status	Monogenic defects diagnosis					Competence evaluation			Transplantable	Clinical outcomes
					Direct mutation detection		Linkage analysis		Result	Chr. exp.	Overall exp.	Prediction		
					Mat.	Pat.	Mat.	Pat.						
1	*CLN3*	AR	Case1E1	5 / B	Het.	Failure	Het.	Het.	Aff.	Norm.	Low	Success	No	Abandoned
			Case1E2	5 / A	WT	Failure	WT	Failure	Unaff.	Norm.	Low	Success	Recom.	**Live birth**
2	*ATXN3*	AD	Case2E1	3 / B	–	WT	–	WT	Unaff.	Norm.	Low	Success	Recom.	Frozen
			Case2E2	1 / A	–	WT	–	WT	Unaff.	Norm.	Low	Success	Recom.	Implantation failure
			Case2E3	3 / B	–	Het.	–	Het.	Aff.	Norm.	Low	Success	No	Abandoned
			Case2E4	3 / B	–	WT	–	WT	Unaff.	Norm.	High	Fail	Alt.	Implantation failure
			Case2E5	5 / A	–	Het.	–	Het.	Aff.	Norm.	High	Fail	No	Abandoned
3	*PRPF31*	AD	Case3E1	6 / B	–	Failure	–	Het.	Aff.	Norm.	High	Fail	No	Abandoned
			Case3E2	2 / B	–	Failure	–	Het.	Aff.	Norm.	Low	Success	No	Abandoned
			Case3E3	4 / B	–	Failure	–	WT	Unaff.	Norm.	Low	Success	Recom.	Frozen
			Case3E4	2 / B	–	Failure	–	Het.	Aff.	Norm.	Low	Success	No	Abandoned
4	*NF1*	AD	Case4E1	3 / B	WT	–	WT	–	Unaff.	Norm.	High	Fail	Alt.	Implantation failure
			Case4E2	4 / B	WT	–	Failure	–	Unaff.	Norm.	Low	Success	Recom.	Frozen
5	*ADA*	AR	Case5E1	1 / A	Het.	WT	Het.	WT	Unaff.	Abn.	High	Fail	Alt.	Frozen
			Case5E2	4 / B	Het.	WT	Het.	WT	Unaff.	Abn.	High	Fail	Alt.	Frozen
6	*EXT1*	AD	Case6E1	2 / A	–	WT	–	WT	Unaff.	Norm.	Low	Success	Recom.	Frozen
			Case6E2	5 / A	–	Het.	–	Het.	Aff.	Norm.	High	Fail	No	Abandoned
7	*NF1*	AD	Case7E1	2 / B	WT	–	WT	–	Unaff.	Abn.	Low	Fail	Alt.	Implantation failure
			Case7E2	4 / C	WT	–	WT	–	Unaff.	Norm.	High	Fail	Alt.	Frozen
8	*PSAT1*	AR	Case8E1	4 / C	Het.		Het.	WT	Unaff.	Norm.	Low	Success	Recom.	**In pregnancy**
			Case8E2	3 / A	WT		WT	WT	Unaff.	Norm.	High	Fail	Alt.	Frozen
			Case8E3	5 / C	Het.		Het.	WT	Unaff.	Norm.	High	Fail	Alt.	Frozen
9	*EXT2*	AD	Case9E1	1 / C	–	Failure	–	Het.	Aff.	Norm.	Low	Success	No	Abandoned
			Case9E2	3 / B	–	Failure	–	WT	Unaff.	Norm.	Low	Success	Recom.	Frozen
			Case9E3	2 / B	–	WT	–	WT	Unaff.	Norm.	High	Fail	Alt.	Implantation failure
			Case9E4	2 / C	–	Failure	–	WT	Unaff.	Abn.	Low	Fail	Alt.	Frozen
			Case9E5	2 / C	–	Failure	–	Het.	Aff.	Norm.	Low	Success	No	Abandoned
10	*PFKP*	AD	Case10E1	2 / B	–	WT	/		Unaff.	Norm.	Low	Success	Recom.	**Live birth**
			Case10E2	5 / B	–	Het.	/		Aff.	Norm.	High	Fail	No	Abandoned
			Case10E3	5 / B	–	WT	/		Unaff.	Norm.	High	Fail	Alt.	Frozen
11	*UBA5*	AR	Case11E1	2 / A	WT	Het.	WT	Het.	Unaff.	Norm.	Low	Success	Recom.	Frozen
			Case11E2	4 / B	WT	Het.	WT	Het.	Unaff.	Norm.	High	Fail	Alt.	Frozen
			Case11E3	4 / A	WT	WT	WT	WT	Unaff.	Norm.	High	Fail	Alt.	Implantation failure
			Case11E4	2 / B	WT (ADO)	WT	Het.	WT	Unaff.	Norm.	High	Fail	Alt.	Frozen
			Case11E5	3 / B	WT (ADO)	WT	Het.	WT	Unaff.	Norm.	High	Fail	Alt.	Frozen
			Case11E6	5 / B	WT	WT	WT	WT	Unaff.	Norm.	Low	Success	Recom.	Frozen
			Case11E7	5 / B	WT	WT	WT	WT	Unaff.	Norm.	High	Fail	Alt.	Frozen
			Case11E8	5 / A	Het.	Het.	Het.	Het.	Aff.	Norm.	High	Fail	No	Abandoned
12	*DYNC1H1*	AD	Case12E1	1 / A	WT	–	WT	–	Unaff.	Norm.	Low	Success	Recom.	**Live birth**
13	*BRCA1*	AD	Case13E1	3 / B	–	WT (ADO)	–	Het.	Aff.	Norm.	Low	Success	No	Abandoned
			Case13E2	2 / C	–	WT	–	Failure	Unaff.	Null	Null	Null	Alt.	**Live birth**
			Case13E3	4 / B	–	Het.	–	Het.	Aff.	Norm.	Low	Success	No	Abandoned
			Case13E4	5 / B	–	WT	–	WT	Unaff.	Norm.	High	Fail	Alt.	Frozen
14	*ATXN3*	AD	Case14E1	2 / A	–	Het.	–	Het.	Aff.	Norm.	Low	Success	No	Abandoned
			Case14E2	3 / C	–	Het.	–	Failure	Aff.	Abn.	High	Fail	No	Abandoned
			Case14E3	3 / B	–	WT	–	WT	Unaff.	Abn.	Low	Fail	Alt.	Frozen
			Case14E4	3 / B	–	WT	–	WT	Unaff.	Norm.	High	Fail	Alt.	**In pregnancy**
			Case14E5	2 / A	–	Het.	–	Het.	Aff.	Norm.	Low	Success	No	Abandoned
			Case14E6	5 / B	–	Het.	–	Het.	Aff.	Abn.	High	Fail	No	Abandoned
15	*LBR*	AR	Case15E1	3 / C	Homo.		/		Aff.	Null	Null	Null	No	Abandoned
			Case15E2	5 / B	Homo.		/		Aff.	Abn.	Low	Fail	No	Abandoned
16	*PMM2*	AR	Case16E1	5 / B	WT	WT	/		Unaff.	Norm.	High	Fail	Alt.	Frozen
			Case16E2	5 / B	WT	Het.	/		Unaff.	Abn.	Low	Fail	Alt.	Frozen
17	*BRCA2*	AD	Case17E1	3 / A	–	Het.	–	Het.	Aff.	Norm.	High	Fail	No	Abandoned
			Case17E2	5 / A	–	WT	–	WT	Unaff.	Norm.	High	Fail	Alt.	Implantation failure
			Case17E3	3 / A	–	Het.	–	Het.	Aff.	Abn.	High	Fail	No	Abandoned
18	*ATXN3*	AD	Case18E1	2 / C	–	Het.	–	Het.	Aff.	Norm.	Low	Success	No	Abandoned
			Case18E2	2 / C	–	Het.	–	Het.	Aff.	Abn.	High	Fail	No	Abandoned
			Case18E3	4 / C	–	Het.	–	Het.	Aff.	Norm.	High	Fail	No	Abandoned
			Case18E4	5 / A	–	Het.	–	Het.	Aff.	Abn.	High	Fail	No	Abandoned
19	*ALPL*	AR	Case19E1	4 / B	WT	Het.	/		Unaff.	Norm.	High	Fail	Alt.	Frozen
			Case19E2	3 / A	Het.	Het.	/		Aff.	Norm.	High	Fail	No	Abandoned
			Case19E3	4 / B	WT	WT	/		Unaff.	Norm.	High	Fail	Alt.	Frozen
			Case19E4	3 / B	WT	Het.	/		Unaff.	Norm.	High	Fail	Alt.	Frozen
20	*BRCA2*	AD	Case20E1	4 / C	WT (ADO)	–	Het.	–	Aff.	Norm.	High	Fail	No	Abandoned
			Case20E2	5 / C	Het.	–	Het.	–	Aff.	Abn.	High	Fail	No	Abandoned
21	*HPRT1*	XLR	Case21E1	5 / C	WT	–	/		Unaff.	Abn.	Low	Fail	Alt.	Frozen
22	*SERAC1*	AR	Case22E1	4 / B	Het.	Het.	/		Aff.	Abn.	High	Fail	No	Abandoned
			Case22E2	3 / B	WT	Failure	/		Unaff.	Abn.	High	Fail	Alt.	Implantation failure
			Case22E3	5 / B	WT	WT	/		Unaff.	Norm.	Low	Success	Recom.	Frozen
			Case22E4	5 / A	WT	WT	/		Unaff.	Abn.	Low	Fail	Alt.	Frozen
23	*HPDL*	AR	Case23E1	4 / C	WT	Het.	WT	Het.	Unaff.	Norm.	Low	Success	Recom.	Frozen
			Case23E2	4 / A	WT	WT	WT	WT	Unaff.	Norm.	Low	Success	Recom.	Implantation failure
			Case23E3	2 / B	Het.	Het.	Het.	Het.	Aff.	Abn.	Low	Fail	No	Abandoned
			Case23E4	5 / B	WT	WT	WT	WT	Unaff.	Abn.	Low	Fail	Alt.	Frozen
24	*MSH6*	AD	Case24E1	4 / A	Het.	–	Het.	–	Aff.	Norm.	Low	Success	No	Abandoned
			Case24E2	5 / A	WT	–	WT	–	Unaff.	Norm.	Low	Success	Recom.	Frozen
25	*EXT1*	AD	Case25E1	4 / C	–	WT (ADO)	/		Aff.	Abn.	Low	Fail	No	Abandoned
			Case25E2	3 / A	–	WT	/		Unaff.	Norm.	High	Fail	Alt.	Frozen
					Pat. (*MLH1*)	Pat. (*MSH2*)								
26	*MLH1*	AD	Case26E1	5 / B	WT	Het.	/		Aff.	Abn.	High	Fail	No	Abandoned
	*MSH2*		Case26E2	5 / A	WT	Het.	/		Aff.	Norm.	Low	Success	No	Abandoned
			Case26E3	5 / B	WT	WT	/		Unaff.	Norm.	Low	Success	Recom.	Frozen

Abbreviations: AR, autosomal recessive; AD, autosomal dominant; XLR, X‐linked recessive; Mat., maternal; Pat., paternal; Het., heterozygote; Homo., homozygote; WT, wild‐type; ADO, allele‐dropout; /, families without performing WES. Null, no prediction results due to low quality of RNA‐seq data. A, represents integral cells with abundant content; B, represents vacuolated cells; C, represents cell fragment; Chr., chromosome; exp., expression; Aff., affected (for AR cases, only embryos carrying both parental mutations are referred to as “affected”); Unaff., unaffected; Norm., normal; Abn., abnormal; Recom., recommendation; Alt., alternative.

Similarly, in case 19, the affected fetus was diagnosed with autosomal recessive infantile hypophosphatasia. Genetic diagnosis identified mutations of c.978_980delCTT (maternal) and c.920C>T (paternal) in the Alkaline Phosphatase, Biomineralization Associated (*ALPL)* gene (Figure S2A and Table S5, Supporting Information). The *ALPL* gene is highly expressed (mean expression level, log_2_(FPKM+1) = 5.86) with no genome coverage (coverage = 0) following WGA (Figure S2B,C, Supporting Information), and four embryos from this family were subjected to RNA‐based diagnosis of this gene. The maternal mutation c.978_980delCTT was directly identified in the mRNA of E2 but was absent in the other 3 embryos (Figure S2D, Supporting Information; Table 1). The paternal c.920C>T was identified in E1, E2 and E4, but not in E3 embryos (Figure S2D, Supporting Information; Table 1).

**Figure 4:**
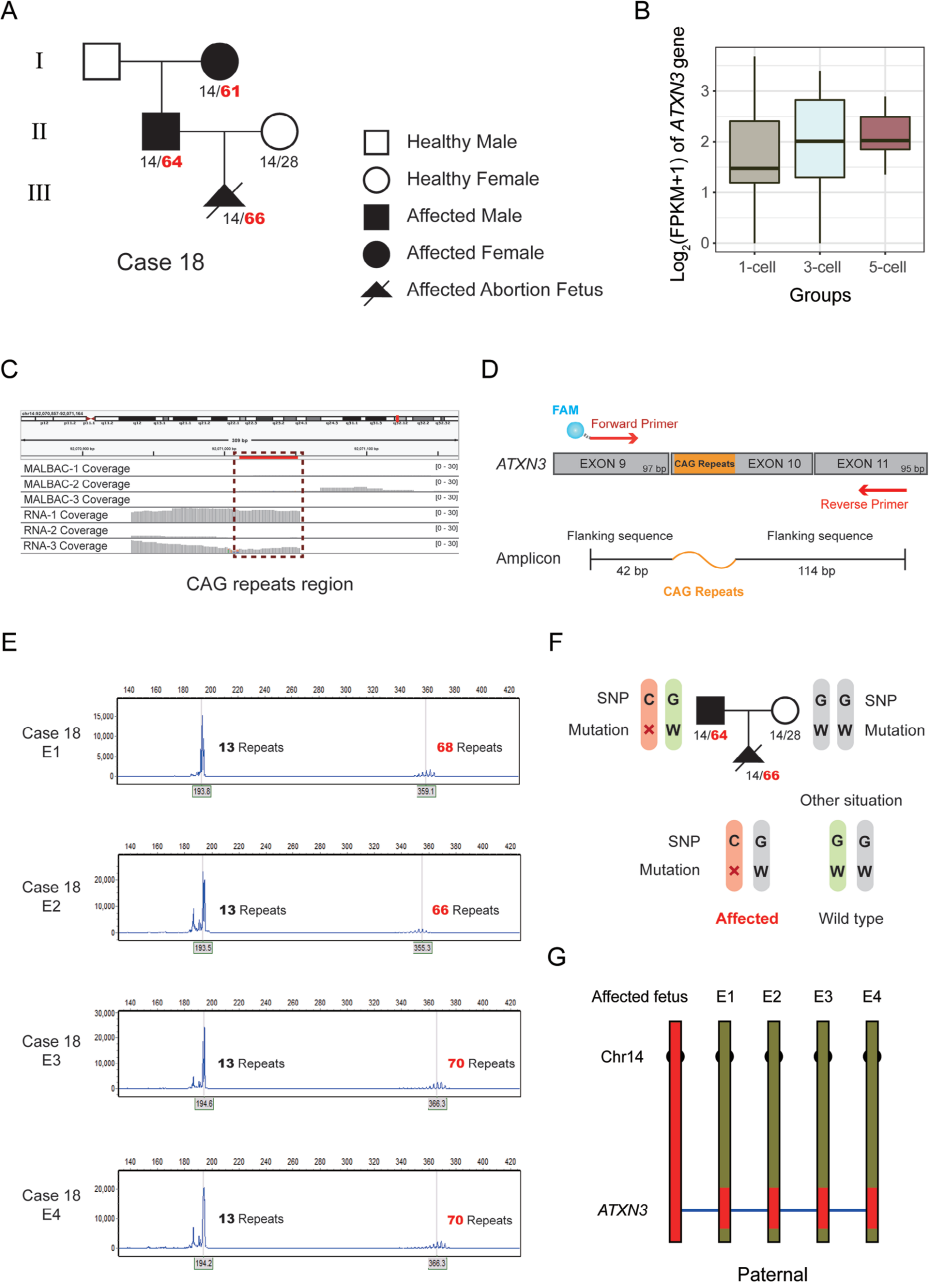
RNA‐based mutation diagnosis for case 18 with autosomal dominant SCA3. A) Pedigrees of the SCA3 family. The filled symbol represents affected individuals, and the open symbol represents wild‐type. The circle, square and triangle indicate female, male and fetus respectively. Diagonal lines represent deceased individuals. The numbers of CAG repeats are shown. The red indicates abnormal expansion. B) *ATXN3* gene expression levels were assessed for three TE groups. The abscissa indicates the 1‐cell, 3‐cell and 5‐cell groups and the ordinate represents the gene expression level united by log_2_ (FPKM+1). C) IGV plot shows coverage of *ATXN3* in DNA and RNA sequencing data from TE cells. The CAG repeat region is indicated by the red dotted box. D) A schematic diagram of primers for CAG repeat detection in *ATXN3* and the composition of the amplicon. The blue gradient globule represents the FAM fluorophore jointed with forward primers. The orange curve represents the dynamic CAG repeats region. E) Direct mutation detection results of 4 embryos in case 18 using capillary electrophoresis following fluorescent PCR. The abscissa indicates the length of amplicon fragments, and the ordinate shows the fluorescence intensity. The red indicates abnormal expansion of CAG repeats. F) SNP analysis schematic of this autosomal dominant case. The symbols “×” indicates the mutation and the letter “W” represents the wild‐type. The SNP base “C” is linked with the mutation and “G” is linked with the wild‐type allele. Embryos carrying C/G inherited parental mutations while those carrying G/G inherited parental wild‐type alleles. G) The SNP analysis results of 4 embryos in case 18 using WES and transcriptome data. SNP markers within 10 Mb upstream/downstream around the mutations were analyzed and are illustrated.

In case 18, the father and the grandmother were diagnosed with autosomal dominant spinocerebellar ataxia type 3 (SCA3) caused by the trinucleotide repeat dynamic mutation in the Ataxin 3 (*ATXN3*) gene, and the fetus was also confirmed to have this abnormal expansion (**Figure** **4**A). The CAG repeats of the father, the grandmother and the fetus were 14/64, 14/61, 14/66 respectively, whereas the CAG repeats of the mother, who was free of the disease, was 14/28 (Figure 4A; Table S5, Supporting Information). The *ATXN*3 gene also exhibited high expression (mean expression level, log_2_(FPKM+1) = 1.92) and no genome coverage (coverage = 0) in the TE (Figure 4B,C), which are ideal for direct RNA mutant detection. Due to the distinct CAG repeats in mutant forms of this gene, the diagnostic approach required specific primers, including fluorophore 6‐FAM forward primers for fluorescence PCR assays (Figure 4D). The CAG repeats in the RNA from each embryo were detected by capillary electrophoresis of the fluorescence PCR reaction products. As shown, all four embryos inherited the abnormal CAG repeat expansion, with the repeat region showing increasing instability (Figure 4E).

### 2.4 Linkage Analysis of Target Genes Based on RNA‐seq Data

Next, we tested whether using RNA‐seq data in linkage analysis would increase the certainty of mutant allele identification. Since SNPs identified in embryonic RNA‐seq data are largely located in exonic regions, we generated bulk WES data for family members and used SNPs in the WES data to construct mutation‐linked haplotypes. The embryo genotypes determined from RNA‐seq data were compared with the mutation‐linked haplotypes to determine the embryonic mutation carrier status. Among the 26 enrolled families, 18 families participated in the linkage analysis study (Table 1). The SNP analysis schematic of case 5 shows a family with autosomal recessive SCID (Figure 3E). For an SNP adjacent to the mutation site, genotypes of the father, mother and proband were C/G, C/G and C/C respectively, suggesting that the C bases link with the mutations. C/C, C/G and G/G indicates affected, carrier and wild‐type embryos, respectively. Following this principle, two embryos of case 5 were analyzed (Figure 3F). The paternal haplotypes of these two embryos (green) were different from those of the proband (red), suggesting that they are free of the paternal mutation. However, the maternal haplotypes of these two embryos (yellow) were concordant with those of the proband (yellow), indicating that they carried the maternal mutation.

Similarly, case 18 was a family with autosomal dominant SCA3. For an SNP adjacent to the mutation site, genotypes of the father, mother and proband are C/G, G/G and C/G respectively, indicating that the C base is linked with the paternal mutation (Figure 4F). There are two embryonic genotypes at this locus, a C/G genotype identical to the affected fetus, and a G/G genotype free of the mutation (Figure 4F). The linkage analysis showed a paternal haplotype in all 4 embryos (red) that was concordant with the affected fetus, implying 4 embryos carried the paternal mutation (Figure 4G).

### 2.5 Embryo Competence Evaluation Based on RNA‐seq Data

**Figure 5:**
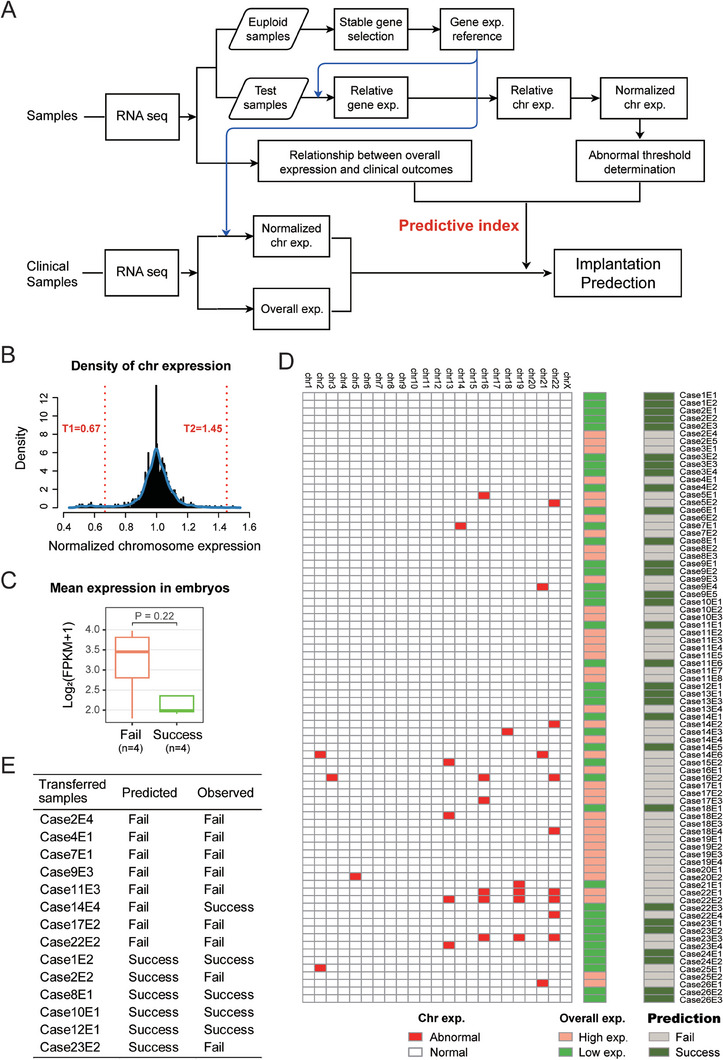
Evaluation of embryo implantation competence. A) Workflow for the developed RNA‐based implantation prediction method in this study. (*Upper aspect*) Establishing the gene reference matrix, calculation the expression in chromosome and overall level respectively, and determining the threshold. (*Lower aspect*) Prediction workflow of implantation competence of all clinical samples. B) Density distribution of normalized chromosome expression in donated samples (Phase I). Chromosomes expressed beyond the normal range (bordered by T1 to T2) are regarded as abnormal. C) Boxplot of the overall expression of transferred samples (Phase I). Failed (orange, n = 4) and successful (green, n = 4) implantations are represented. P = 0.22. D) Schematic diagram of implantation prediction by our RNA‐based method in clinical samples (Phase II). The left heatmap shows the chromosome expression. The red and white boxes represent abnormal and normal chromosomes, respectively. The middle heatmap shows the overall high (orange) and low (green) expression. The right heatmap shows the predicted outcome from implantation. Grey boxes (embryos with abnormal chromosome expression and/or high overall expression) represent embryo predicted to fail implantation; dark green boxes (embryos without abnormal chromosome expression and with low overall expression) represent embryos that will show successful implantation. E) The predicted and observed clinical outcomes of transferred embryos (Phase II).

Embryo competence is central to a successful implantation event, and this requires appropriate gene expression. We reasoned that abnormal gene expression may reflect embryo competence and could be predictive for embryo implantation outcomes. To address this possibility, we developed an RNA‐based approach (**Figure** **5**A). Briefly, we studied the expression patterns of normal embryos at the level of chromosomal expression, as well as overall expression. At the chromosome level, the relative expression of each chromosome for each sample was calculated. As shown in Figure 5B, the relative expression of genes from the overwhelming majority of chromosomes were normally distributed, although some chromosomes showed abnormally high or low expression. We hypothesized that the occurrence of these abnormal expression features for chromosomes will lead to the failure of embryo implantation. From our analysis of the overall expression levels of embryos, we found that overall high expression occurred in embryos that failed to implant (n = 4) (Figure 5C). Therefore, in our model, according to the overall expression level, the embryos were divided into high expression and low expression groups.

Combining the information of these two levels, we next reasoned that those embryos with normal chromosomal expression and overall low expression could identify these to be successfully implanted. That is, embryos with abnormal chromosomal expression or overall high expression are regarded as low developmental competence embryos. From this, the competence of clinical embryos (that is, to be selected for implantation) was evaluated (Figure 5D). A total of 30 among 80 qualified embryos were predicted to be successful implantations (Table 1).

### 2.6 Clinical Results and Outcomes

We diagnosed the status of genetic mutations by direct mutation detection and linkage analysis and evaluated the competence of the 82 embryos based on the transcriptome simultaneously (Table 1). In parallel, we obtained routine PGT results for these embryos (Table S6, Supporting Information). In terms of our mutation diagnosis from the transcriptome, the success and accuracy rates of direct mutation detection were 90% (100/111) and 95% (95/100), whereas the success and accuracy rates of linkage analysis were 95% (76/80) and 100% (76/76) (Table 1). Among 82 embryos from these 26 families, 48 embryos were identified to be healthy and free of mutations that could be selected for transfer (Table 1).

**Figure 6:**
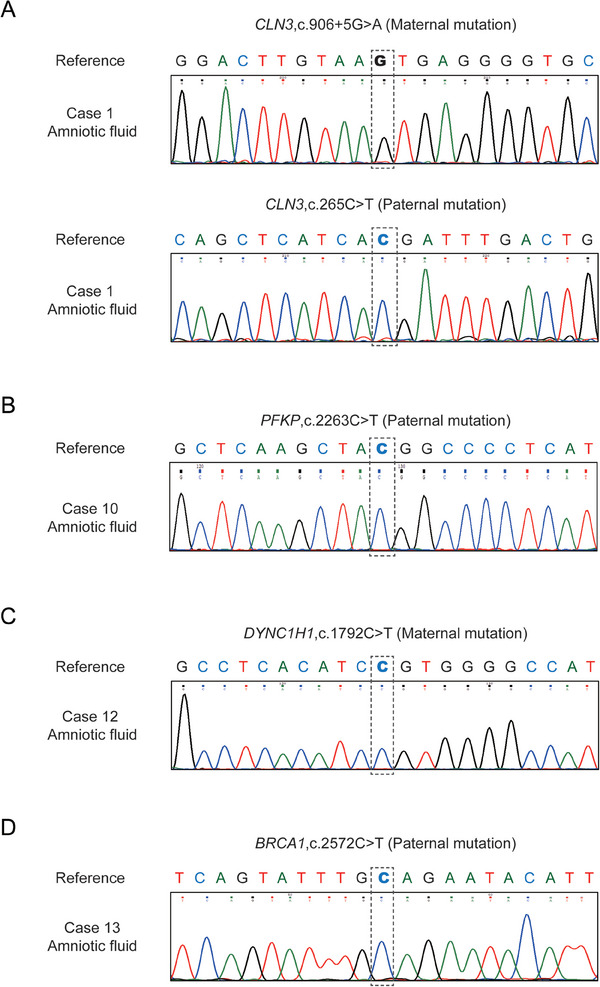
Verification of RNA‐based PGT‐M results through prenatal diagnosis. A–D) Mutation detection of amnion fluid cells using Sanger sequencing of specific PCR products for case 1, case 10, case 12 and case 13 respectively. Grey dotted lines show the mutation loci.

The clinical outcomes and the pregnancy outcomes are summarized in Table 1. As shown, fifteen embryos from 14 families were transferred and six embryos were successfully implanted (Table 1). Comparing the predicted and observed clinical outcomes of these 14 transferred embryos (except for Case12E2 that lacked a prediction result), our prediction system showed high accuracy, with 11 (78.6%) predictions that were correct (Figure 5E). Two embryos were at the 8‐week and 11‐week stages of pregnancy (case 8 and 14, respectively) and four (case 1, 10, 12, 13) produced healthy neonates. These four families with neonates have completed prenatal amniocentesis diagnosis at 20 weeks of gestation using genomic DNA from the cultured amnion fluid cells, and the results are consistent with our RNA‐PGT diagnosis (**Figure** **6**A–D).

We performed differential expression analysis of embryos from successful and failed implantations to further identify potential marker genes related to implantation. From this, we identified thirty‐one differentially expressed genes (DEGs) between the two groups, including 3 down‐regulated and 28 up‐regulated genes in the failure group (Figure S3A, Supporting Information). Specifically, *C19orf53*, *FIGNL2* and *HSF2BP* were downregulated in the failure group, and these genes were categorized to be involved in tumorigenesis, cytoplasmic microtubule organization and maintenance of embryonic stem cell state, respectively (Figure S3B, Supporting Information). A further 28 genes were found to be upregulated in the failure group, with over 50% of them barely expressed in the success group (Figure S3C, Supporting Information). Among them, fifteen genes are involved in cell cycle control, tumorigenesis, immunity response, mitochondrial energy metabolism and immunity‐apoptosis signaling. All of these biological mechanisms are highly relevant to the process of embryo implantation (Figure S3C, Supporting Information).

## 3 Discussion

Since the first clinical application of PGT, many families have been able to have healthy children that are free from an inheritable genetic disease. While PGT analysis began with a method to analyze only two copies (single cell) of DNA before WGA, the uneven coverage and ADO associated with these DNA amplification methods represent significant confounds that affect the reliability of this genetic diagnostic approach. Here, we reasoned that multiple copies mRNA would offer a more sensitive and accurate means to detect mutations in embryos. Starting with a transcriptome‐level assessment of donated blastocysts, we then developed and tested an RNA‐based PGT method, applying it to 26 monogenic disorder families so as to diagnose 82 embryos. Given that our RNA‐based PGT method is also informative for assessing embryo competence, this approach could be valuable for clinical implantation guidance.

Accurate diagnosis of mutations in embryos is the primary consideration for PGT‐M. Compared with two copies of target genes in the single‐cell genome, abundant mRNA copies are present in the single‐cell transcriptome and could improve diagnostic sensitivity and reliability. Theoretically, our method is appropriate for all pathogenic genes that are expressed in TE. Specifically, RNA‐based PGT has greater advantage when applied to screen for mutations in unbiased highly expressed genes with low genomic coverage and depth after WGA (Figure 2F; Table S4, Supporting Information). For instance, the *ATXN3* gene, in which abnormal expansion of CAG trinucleotide repeats will cause SCA3. The CAG repeats are GC rich and difficult to amplify on the genome level. Using the PGT‐M method based on the transcriptome, we directly detected the CAG repeats in embryos from three SCA3 families (Figure 4E and Table 1), which has not been previously reported. This suggests that our RNA‐based PGT method has the potential for clinical application to screen embryos for such monogenic disorders.

Successful implantation signals the initiation of pregnancy, and a viable embryo is essential for proper implantation and normal development. Recent studies suggest that TE is closely related to embryo implantation and development, and gene expression of TE can reflect the embryo competence.^[^ ^21^, ^22^, ^24^, ^25^ ^]^ Therefore, we assessed embryo competence and further predicted its implantation potential using TE transcriptome. Our predictive system achieved an accuracy of up to 78.6% (11/14) when identifying embryos that would be successful or unsuccessful for implantation (Figure 5E). Indeed, our approach correctly identified seven out of eight (87.5%) embryos that failed to implant. Of the six embryos that were predicted to implant successfully, four embryos met such expectations (66.7%). Guided by these findings, it would appear that our model is more accurate for predicting “Fail” embryos than embryos that will show “Success” in implantation. One explanation is that the successful implantation requires the supporting of multiple factors, including a permissive embryo and uterine microenvironment, and disruptions to any of these factors could lead to implantation failure.

Our differential gene expression analysis of embryos from successful and failed implantations identified 31 genes associated with important biological processes and signaling pathways involved in implantation. The vast majority (28/31) of DEGs exhibited up‐regulation in failed implantations which is consistent with the relationship between overall expression level and implantation potential in our prediction model. Among the up‐regulated genes, four of these (*RIPK1*, *USP15*, *SMAD4* and *SH3RF1*) are involved in the immunity‐apoptosis signaling pathway (Figure S3C, Supporting Information). Receptor‐interacting serine/threonine‐protein kinase 1 (*RIPK1*) is a primary regulator of the cell fate decision.^[^ ^28^ ^]^ High expression of *USP15* protects *RIPK1* from degradation through deubiquitylation, and upregulation of *RIPK1* could phosphorylate and activate downstream *SMAD4*.^[^ ^29^, ^30^ ^]^ The pro‐apoptotic protein *SH3RF1* can respond to the upstream signal from *SMAD4* and induce apoptosis and cell death, which may trigger implantation failure.

We recognize that our RNA‐based PGT‐M method has some limitations. First, our approach to mutation detection is based on sampling mRNA in embryos and, as such, pathogenic variants in introns with unknown effects on transcripts cannot be directly detected. Nevertheless, such variants can be resolved by RNA‐based linkage analysis. As an adjunct method for double verification, the resolution of RNA‐based linkage analysis is not as precise as routine DNA‐based PGT‐M. Furthermore, our implantation prediction system is a quantitative approach based on the expression level of the transcriptome. Due to fluctuations in gene expression levels, our RNA‐based PGT‐M and embryo implantation prediction system requires biopsied cells with high quality. In addition, although we have assessed parental expression bias at the mRNA level using SNP heterozygosity, there are still few specific genes that may have differences of parental expression within the individual, resulting from individual‐specific genetic or epigenetic patterns.

In summary, we have developed a new RNA‐based PGT approach to simultaneously detect genetic mutations in embryos and assess their suitability for implantation. This method is suitable for highly expressed and low‐biased genes, especially those with low genomic coverage and depth after WGA in routine PGT‐M. In addition, the embryos competence assessment was also performed and showed a higher accuracy than routine PGT. We anticipate such an approach will be broadly implemented in the clinic to improve the detection of inheritable genetic mutations that cause lifelong disability and shortened lifespan in children and the young.

## 4 Experimental Section

### Sample Collection for Feasibility Assessment

This study was approved by the Reproductive Medicine Ethics Committee of Peking University Third Hospital (2019SZ‐085). In the phase of feasibility assessment, 20 couples were enrolled, and extensive informed consents were signed by participants when they authorized biopsy and donation. A total of 19 donated whole blastocysts and 21 biopsied TE materials from 21 blastocysts were obtained (Table S1, Supporting Information).

For donated whole blastocysts, after removing the zona pellucida using 5‰ hydrochloric acid, blastocysts were digested into single cells with mixed enzymes (accutase and pancreatic enzyme in equal proportions) and incubated at 37 °C for 30 min. Next, TE cells from each blastocyst were divided into three groups: 1‐cell, 3‐cell and 5‐cell groups. The TE cells from each of the 3 groups were collected in RNA lysis buffer for subsequent single‐cell RNA‐seq. The remaining cells of each blastocyst were collected in DNA lysis buffer for single‐cell DNA‐seq (Figure 1 and Table S1, Supporting Information).

Biopsied TE samples were digested into single cells with mixed enzymes (accutase and pancreatic enzyme in equal proportions) and incubated at 37 °C for 30 min. The single cells of each biopsied TE materials were divided into two portions, ≈3 cells collected for single‐cell RNA‐seq, and remaining ≈3 cells collected to perform single‐cell DNA‐seq as a comparison (Figure 1 and Table S1, Supporting Information).

### Recruitment of Monogenic Disorder Couples

From 2020 to 2022, 26 couples were recruited into our study at the Reproductive Center of Peking University Third Hospital, and all couples provided written informed consent (2019‐SZ‐085). The genetic information for these couples was shown in Table S5 (Supporting Information). Among the 26 families were 18 monogenic disorders (including autosomal dominant, autosomal recessive and X‐linked recessive genetic disorders), referring to 22 pathogenic genes. The types of pathogenic variants include point mutation, splicing mutation, frameshift mutation (small insertion/deletion) and trinucleotide repeat dynamic mutation (Table S5, Supporting Information).

### Clinical Blastocyst Biopsy and Sample Collection

In this study, each monogenic disorder couple underwent 1 PGT cycle, and a total of 377 matured metaphase II (MII) stage oocytes were collected (Table S5, Supporting Information). After intracytoplasmic sperm injection (ICSI), 211 mature oocytes were fertilized. Approximately 5–6 days after fertilization, 82 embryos developed to the blastocyst stage and reached the embryo biopsy standard (Table S5, Supporting Information). Laser‐guided TE biopsies were performed to obtain 5–8 TE cells from each sampled embryo. Biopsied materials were digested into single cells with mixed enzymes (accutase and pancreatic enzyme in equal proportions) and incubated at 37 °C for 30 min. The single cells of each blastocyst were divided into two portions, ≈3 cells collected for RNA‐based genetic testing, and another ≈3 cells collected to perform routine DNA‐based PGT as a comparison (Figure 1). The quality of samples was recorded as 3 grades (Table 1), with “A” representing integral cells with abundant contents, “B” representing vacuolated cells and “C” representing cell fragments.

### Single Cell RNA‐seq

Three groups of TE samples from 19 donated blastocysts and biopsied samples of 21 blastocysts in phase I, and 82 biopsied samples of clinical blastocysts in Phase II were used for single cell RNA‐seq experiments (Figure 1). Sufficient cDNA from lysed TE samples was obtained for each sample using a Smart‐seq2 method.^[^ ^31^ ^]^ Specifically, cells were lysed, and RNA released in a relatively hypotonic lysis buffer without interfering the following reverse transcription (RT) reaction. Next, the RT reaction, also called the first‐strand reaction, was performed at 42 °C for 90 min using tailed oligo‐dT oligonucleotides that can trigger this reaction on polyadenylated RNA sequences. After the first‐strand reaction, the cDNA is amplified using 18 PCR cycles to generate enough material for the following steps. Next, libraries were prepared using NEBNext Ultra II DNA Library Prep Kit for Illumina (New England Biolabs, Inc.) and NEBNext Multiplex Oligos for Illumina (New England Biolabs, Inc.). The quantity and quality of libraries were both detected and assessed using Thermo Fisher Qubit fluorometer and Agilent Fragment Analyzer. All samples were sequenced on the Illumina NovaSeq platform (USA).

### Single Cell DNA‐seq and WES

All corresponding samples both in phase I and II collected in DNA lysis buffer were subjected to single cell DNA‐seq. Whole‐genome DNA was amplified using a commercial MALBAC amplification kit (Yikon Genomics Inc., China). Libraries were prepared using NEBNext Ultra II DNA Library Prep Kit for Illumina (New England Biolabs, Inc.) and NEBNext Multiplex Oligos for Illumina (New England Biolabs, Inc.). Next, the quantity and quality of libraries was detected and assessed using Thermo Fisher Qubit fluorometer and Agilent Fragment Analyzer. The libraries were sequenced on the Illumina NovaSeq platform (USA).

WES was performed for 18 of the 26 recruited families (Figure 1). Genomic DNA was extracted from 200 µL of peripheral blood, using a Qiagen DNA Blood Mini kit (Qiagen, Inc.) following the manufacturer's protocol. Libraries were subject to target capture using SureSelect Human All Exon V6 (Agilent) followed by sequencing to 100× depth on the Illumina NovaSeq platform.

### RNA‐based Detection of Pathogenic Variants

Following reverse transcription of RNA from samples, the resultant cDNA samples were used for detecting pathogenic variants as follows. For SCA3 families (case 2, 14 and 18) that have a genetic condition caused by abnormal expansion of CAG trinucleotide repeats in the *ATXN3* gene, capillary electrophoresis following fluorescent PCR with specific 6‐FAM‐labeled primers for PCR amplicons was used (Figure 4D). The amplicons contain two parts, an immobilized 156 bp flanking sequence and a variable CAG repeat sequence (Figure 4D). GeneMarker (version 2.2.0) was used to measure the amplicons length and the formula (amplicon length – flanking length)/3 was used to calculate the number of CAG repeats. Mutation detection of other families was accomplished using PCR with specific primers targeting the mutation sites followed by Sanger sequencing. The primer design takes full‐length mRNA transcripts as a reference, because the cDNA used for PCR detection was reverse transcribed from mRNA. As the mRNA only contains exons, it complies with the general principles of primer design, where intron regions are excluded. The primers used in this study were listed in Table S7 (Supporting Information).

### Processing of RNA‐seq Data

Raw, pair‐end sequencing reads were trimmed by Trim_Galore (version 0.6.6) with parameters as follows: –quality 20 –phred33 –stringency 3 –length 36. The trimmed reads were mapped to hg38 reference genome (UCSC) with default parameters using RSEM aligner (version 1.3.3).^[^ ^32^ ^]^ The count of each gene was quantified by the featureCounts program with following parameters: ‐p ‐t exon ‐g gene_id.^[^ ^33^ ^]^ Fragments per kilobase per million mapped reads (FPKM) was obtained by RSEM.

TE cells expressed (FPKM>1) an average of 7000 genes, 8000 genes, and 8800 genes in the 1‐cell, 3‐cell, and 5‐cell groups, respectively, and the overwhelming majority (54/57, 94.7%) of the TE samples expressed over 5000 genes (Figure 2A,B). The number of expressed genes could reflect the cell quality. To avoid the influence of low‐quality samples, we excluded samples that expressed fewer than 5000 genes as detected in the subsequent analysis.

### SNP Calling by RNA‐seq Data

For RNA‐seq data of embryos, bam files generated by RSEM were used to call SNPs. Duplicated reads were removed, and only uniquely mapped reads were retained via Samtools (version 1.5). GATK variant‐calling pipeline suitable for RNA‐seq data was applied to call SNPs. SNPs within 10 Mb upstream/downstream of the mutation site were filtered by GATK VariantFiltration.^[^ ^34^, ^35^ ^]^

### RNA‐based Linkage Analysis

Linkage analysis was performed based on embryonic RNA‐seq data and the WES data of the participating family members. SNPs within 10 Mb upstream/downstream of the mutation site in the WES data (bulk WES data for family members) were phased to construct the mutation‐linked haplotypes, and the embryonic haplotypes constructed by RNA data were compared with the mutation‐linked haplotypes to identify the mutation carrier status of embryos.^[^ ^36^ ^]^

### RNA‐based Chromosomal Expression Calculation

It was reasonable that abnormal expression was indicative of gene dysfunction, which may affect embryo competence. Therefore, the gene expression pattern of embryos could be a predictor for implantation outcomes. At first, the chromosomal expression patterns of embryos was studied.

The RNA‐based chromosomal expression calculation includes three steps, part of which draws on the algorithm of inferCNV.^[^ ^37^, ^38^, ^39^, ^40^, ^41^ ^]^ Samples diagnosed as normal diploid by routine PGT‐A were selected to generate normalization factors. Specifically, the stability of each gene was measured by coefficient of variation (CV) in euploid blastocysts (Figure S1B, Supporting Information). The unexpressed genes and 20% most unstable genes were excluded. Then the average expression level of each gene was calculated as normalization factors. Gene expression of test samples was divided by normalization factors to form a relative expression matrix. For each chromosome, expression levels across genes were summed and then averaged by gene numbers to generate relative expression values. Within each sample, the relative expression values of the chromosomes are converted to a median of 1, as normalized chromosome expression. The details were as follows:

First, the normalization factor for each gene was calculated by averaging the expression of the gene in diploid samples:

(1) $$\left[\begin{array}{cc} Gene\;Name & Mean\;exp\\ gene_{1} & e_{1}\\ gene_{2} & e_{2}\\ \vdots & \vdots \\ gene_{k} & e_{k} \end{array}\right]$$

where normalization factor *e_k_* represents the average expression of gene *k* in diploid TE samples.

Subsequently, for each sample *i*, the raw expression level of gene *k* on chromosome *j* (*raw*exp_*i*,*j*, *k*_) was normalized by the above normalization factor as follows:

(2) $$\mathrm{ex}p_{i,j,k}=\frac{raw\mathrm{ex}p_{i,j,k}}{\begin{array}{c} e_{k}\\ \; \end{array}}\;$$

where exp_*i*,*j*, *k*_ is the normalized expression of gene *k* on chromosome *j* in sample *i*.

Next, the expression of chromosome *j* in sample *i* is calculated by averaging the normalized expression of all genes within the chromosome:

(3) $$\mathrm{ex}p_{i,j}=\frac{\sum_{k=1}^{k=n_{j}}\mathrm{ex}p_{i,j,k}}{\begin{array}{c} n_{j}\\ \; \end{array}}\;$$

where exp_*i*,*j*_ denotes the expression of chromosome *j* in sample *i*, and *n_j_* represents the total number of genes in chromosome *j*.

Finally, the chromosome expressions of each sample were centered to 1 by dividing by the median chromosome expression of the sample:

(4) $$EX\;\;\;P_{i,j}=\;\frac{\mathrm{ex}p_{i,j}}{median\left(\mathrm{ex}p_{i,j}\right)}$$

where *EXP*_*i*,*j*_ denotes the final chromosome expression of chromosome *j* in sample *i*.

It was reasonable that the expression levels of most chromosomes in normal diploids adhere to a normal distribution. However, genomic aberrations or chromosome dysfunction could lead to expression levels beyond a normal range. Hence, a “local minimum” value in the distribution plot to delineate the thresholds for “normal” and “abnormal” expression levels was utilized. As shown in Figure 5B, 0.67 represents the local minimum on the left side of the normal distribution curve. Chromosomes with expression levels below this threshold exhibited extremely low expression. Conversely, 1.45 serves as the local minimum on the right side of the distribution. Chromosomes with expression levels surpassing this threshold were aberrantly overexpressed. Abnormal chromosomal expression was anticipated to lead to implantation failure.

### RNA‐based Overall Expression Calculation

Suitable gene expression of a preimplantation embryo is central to its competence and subsequent successful implantation. From our analysis of the overall expression levels of transferred embryos, it was found that successfully implanted embryos showed a relatively low overall expression, while embryos that failed to implant showed a relatively high overall expression (Figure 5C). Therefore, overall expression level was selected as a further indicator to predict the embryo's implantation ability. Genes expressed in more than two samples among eight transferred embryos (four successful implantations B2, B7, B13 and B16, and four failed implantations B5, B9, B11 and B19) were selected (Table S1, we utilized). In subsequent analyses, expression levels of these genes were summed in each clinical sample. The half of the sample with higher expression was considered to have low implantation potential (Figure 5C).

### Differential Analysis Between Implantation Success and Failure Groups

Differential expression analysis of embryos was performed from successful (n = 5) and failed (n = 6) implantations. Among six successful implantations, Case13E2 was discarded owing to low quality of RNA‐seq data (Table 1). Among nine failed implantations, two embryos (Case2E2 and Case23E2) predicted “Success” were discarded and one embryo (Case22E2) was excluded due to abnormalities of chromosome expression (Table 1). The differentially expressed genes (DEGs) between successful and unsuccessful implanted embryos were identified by the R package DESeq2 (version 4.2.2). DEGs were selected based on a padj cutoff of 0.05. Heatmaps and boxplots were drawn with pheatmap and ggplot2 function in R, respectively.

### Processing of Single Cell DNA‐seq and WES Data

The raw single cell DNA‐seq data of blastocysts and WES data of family members were trimmed by Trim_Galore (version 0.6.6). The trimmed reads were mapped to hg38 reference genome (UCSC) using BWA‐MEM (version 0.7.17) with default parameters. PCR duplications and non‐uniquely mapped reads were removed by Samtools (version 1.5).

### SNP Calling by Single Cell DNA‐seq and WES Data

GATK Best Practices pipeline was used to call SNPs in single cell DNA‐seq and WES data. The raw SNPs were filtered using GATK VariantFiltration.

### Copy Number Analysis by Single Cell DNA‐seq Data

Aneuploidy analyses were conducted as previously described.^[^ ^42^ ^]^ Briefly, the mapped reads were counted with a window of 1 Mb using readCounter software. R package HMMcopy was then utilized to detect aneuploidies.^[^ ^43^, ^44^ ^]^

### Statistical Analysis

Statistical analyses were conducted using R. In instances where comparisons were made between two groups, such as assessing the overall expression levels between the failure and success groups, an unpaired T‐test was utilized. The graphical representations depicted mean values ± standard deviation (SD), with the number of samples for each statistical analysis detailed in the figure captions. Significance was established at a p‐value of < 0.05.

### Ethics Approval Statement

This study was approved by the Reproductive Medicine Ethics Committee of Peking University Third Hospital (2019SZ‐085). Patients were extensive informed, and consents were signed by participants.

## Conflict of Interest

The authors declare no conflict of interest.

## Author Contributions

Y.W., Y.L., and X.Z. contributed equally to this work. J.Q., L.Y., Z.Y. performed conceptualization, Y.W., Y.L., X.Z., M.Y., Y.L., N.W., C.L., Y.K., Y.L., J.H., J.J. performed data curation, J.Q., L.Y., Z.Y. performed funding acquisition, Y.W., Y.L., X.Z. performed investigation, Y.W., Y.L., X.Z., M.Y., Y.L., N.W., C.L., Y.K., Y.L., J.H., J.J. performed methodology, J.Q., L.Y., Z.Y., Y.W., Y.L. performed project administration, J.Q., L.Y., Z.Y., C.C.L.W. performed supervision, Y.W., Y.L. performed validation, Y.W., Y.L., X.Z. performed visualization, Y.W., Y.L., X.Z. wrote the original draft, Y.W., Y.L., X.Z., J.Q., L.Y., Z.Y. reviewed and edited the manuscript.

## Supporting information

Supporting Information

Supporting Information

## Data Availability

The data that support the findings of this study are available from the corresponding author upon reasonable request.
