# Supplementary material for: Concurrent Preimplantation Genetic Testing and Competence Assessment of Human Embryos by Transcriptome Sequencing
Source: Adv Sci (Weinh). 2024 Jun 20;11(32):2309817. doi: 10.1002/advs.202309817 (PMC11348190; doi:10.1002/advs.202309817)
Supplement: Supplementary file 1 — Supporting Information [file ADVS-11-2309817-s001.docx]

**Supporting Information**

**Title:** Concurrent Preimplantation Genetic Testing and Competence Assessment of Human Embryos by Transcriptome Sequencing

**Authors:** *Yuqian Wang†, Ye Li†, Xiaohui Zhu†, Ming Yang, Yujun Liu, Nan Wang, Chuan Long, Ying Kuo, Ying Lian, Jin Huang, Jialin Jia, Catherine C. L. Wong, Zhiqiang Yan*, Liying Yan* and Jie Qiao**

† Joint First Author, * Corresponding author

**Supplementary Figures**

**
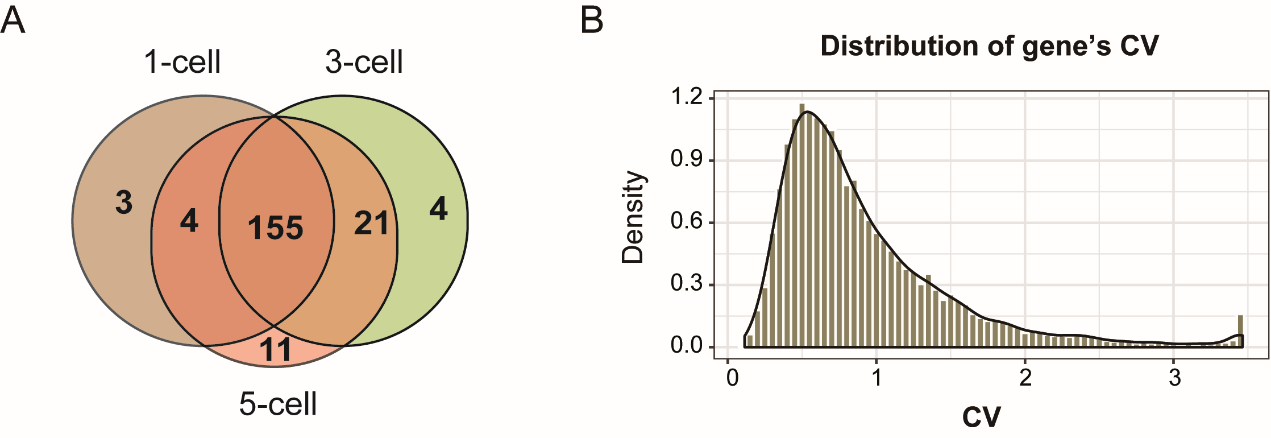
**

**Figure S1.** Gene expression features of TE cells in donated blastocysts.

A) Venn diagram showing highly expressed genes (log_2_(FPKM+1)>1.5) in 1-cell, 3-cell and 5-cell groups. B) Distribution of coefficient of variation for genes analyzed. When calculating chromosomal expression, the gene reference matrix was constructed using the top 80% genes with the smallest CV value.

**
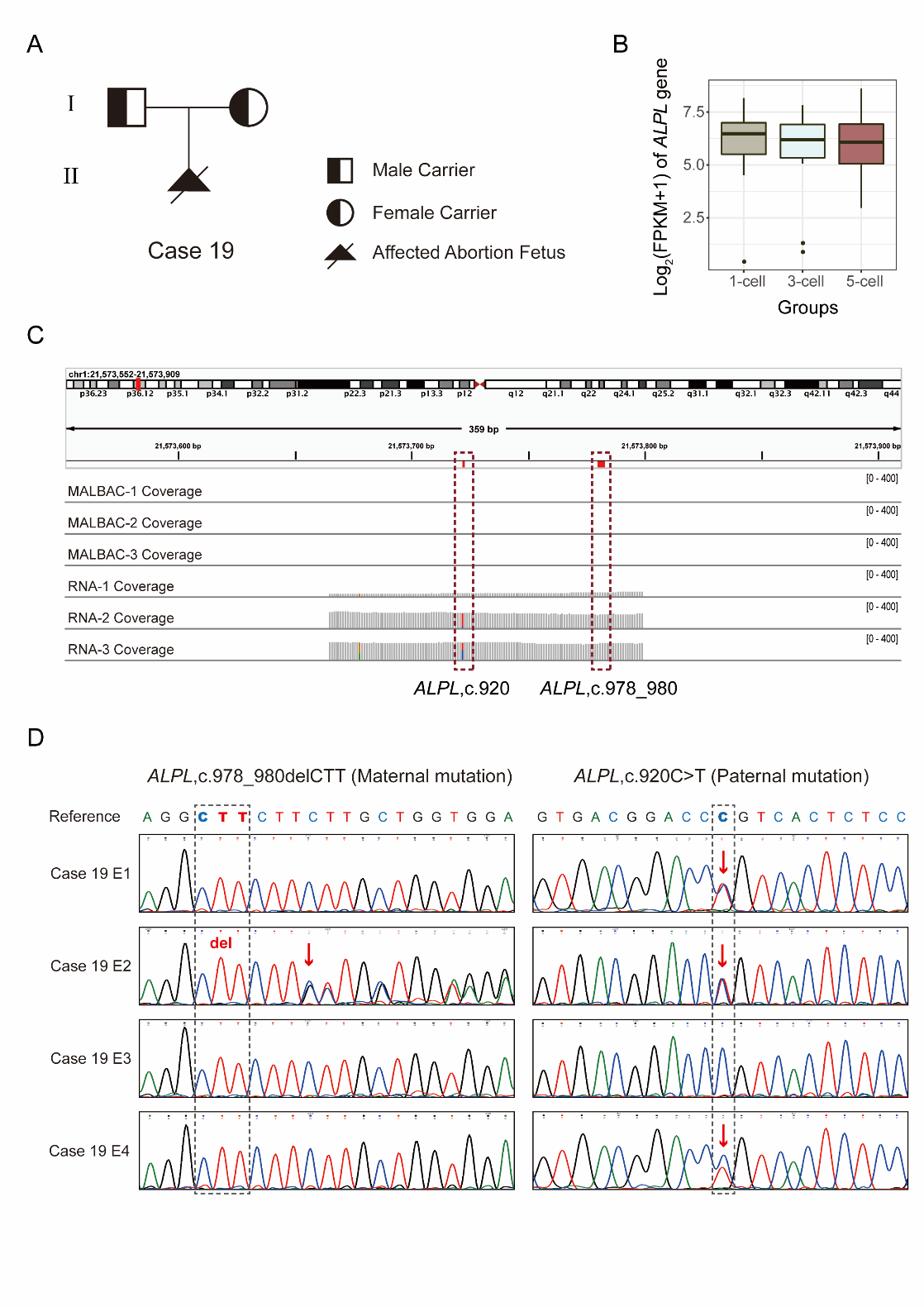
**

**Figure S2.** RNA-based mutation diagnosis for case 5 with autosomal recessive infantile hypophosphatasia.

A) Pedigrees of the infantile hypophosphatasia family. The filled symbol represents the affected individual and the half-filled symbol represents carriers of this disease. The circle, square and triangle indicate female, male and fetus, respectively. The diagonal line represents a deceased individual. B) *ALPL* gene expression levels were assessed for three TE groups. The abscissa indicates the 1-cell, 3-cell and 5-cell groups and the ordinate represents the gene expression level united by log_2_(FPKM+1). C) IGV plot shows the coverage of *ALPL* in DNA and RNA sequencing data from TE cells. Mutation loci are indicated by red dotted boxes. D) The direct mutation detection results of these 4 embryos through Sanger sequencing following PCR amplification from RNA. Dotted boxes show the mutation loci and the red arrows indicate the mutations.

**
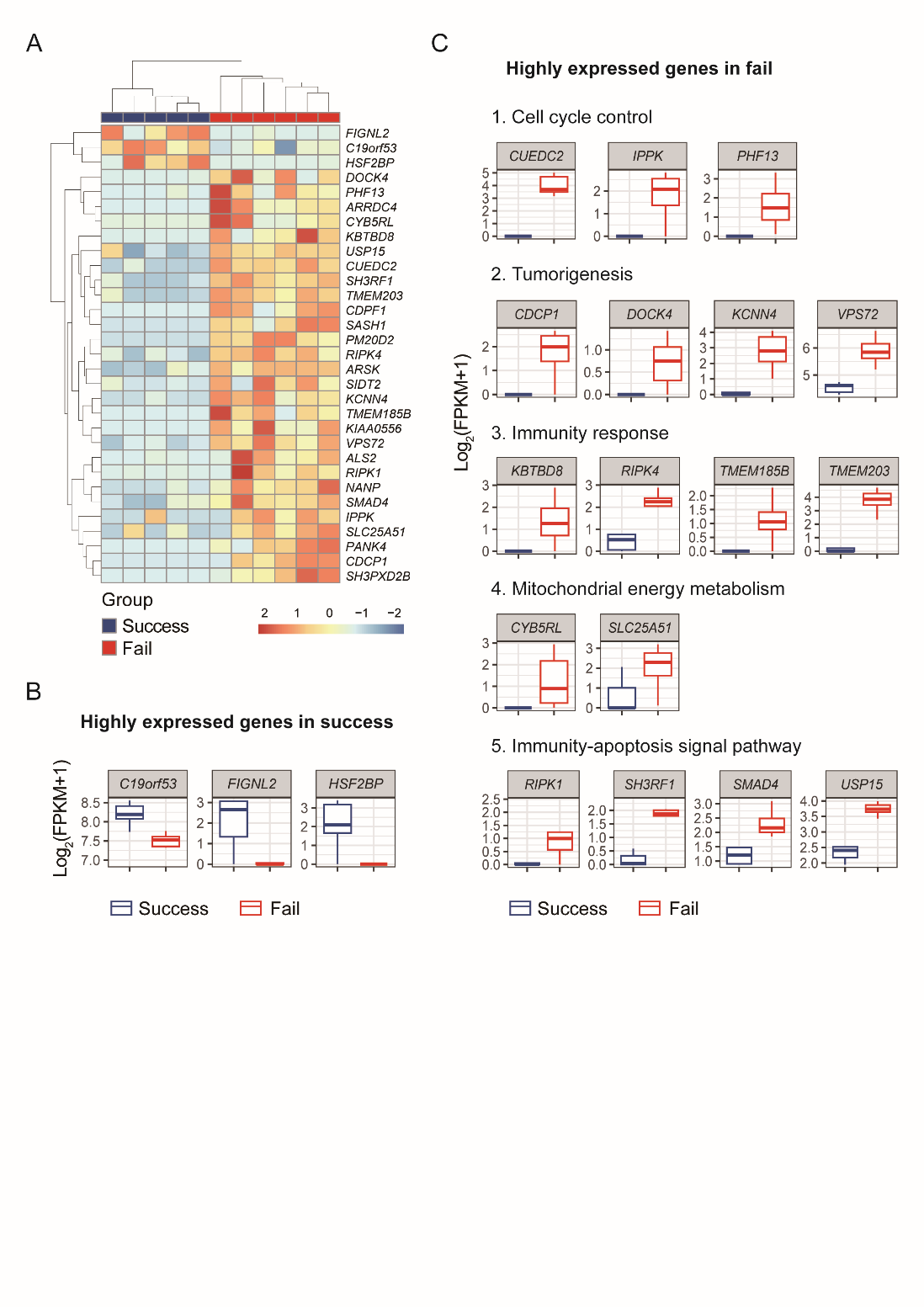
**

**Figure S3.** Differential expression analysis identified potential genes associated with implantation.

A) Heatmap of DEGs for successful (n=5) and failed (n=6) implantation embryos. B) Boxplot shows down-regulated DEGs in failed implantations. C) Boxplot shows up-regulated DEGs in failed implantations.
